# A review of triage accuracy and future direction

**DOI:** 10.1186/s12873-018-0215-0

**Published:** 2018-12-20

**Authors:** Hon Lon Tam, Siu Fung Chung, Chi Kin Lou

**Affiliations:** 1 Kiang Wu Nursing College of Macau, Est. Repouso No. 35, R/C, Macau, S.A.R. China; 2 Flinders University, Sturt Road, Bedford Park, 5042 Adelaide, South Australia; 3 City University of Macau, Avenida Padre Tomás Pereira Taipa, Macau, S.A.R. China

**Keywords:** Triage accuracy, Emergency nursing, Training, Monitoring, Collaboration

## Abstract

**Background:**

In the emergency department, it is important to identify and prioritize who requires an urgent intervention in a short time. Triage helps recognize the urgency among patients. An accurate triage decision helps patients receive the emergency service in the most appropriate time. Various triage systems have been developed and verified to assist healthcare providers to make accurate triage decisions. The triage accuracy can represent the quality of emergency service, but there is a lack of review studies addressing this topic.

**Methods:**

A literature search was conducted in four electronic databases where ‘emergency nursing’ and ‘triage accuracy’ were used as keywords. Studies published from 2008 January to 2018 August were included as potential subjects. Nine studies were included in this review after the inclusion and exclusion criteria were applied.

**Results:**

Written case scenarios and retrospective review were commonly used to examine the triage accuracy. The triage accuracy from studies was in moderate level. The single-center studies which held better results than those from multi-center studies revealed the need of triage training and consistent training between emergency departments.

**Conclusions:**

Regular refresher triage training, collaboration between emergency departments and continuous monitoring were necessary to strengthen the use of triage systems and improve nurse’s triage performance.

## Background

Emergency department (ED) in a hospital is to provide the immediate interventions for those with urgent and critical needs. After registration of seeking emergency service, triage is the first encounter between healthcare providers and patients [1]. The function of the triage in a hospital is to identify and prioritize those with the most urgent needs to use the emergency service first [2, 3]. An accurate triage decision is a correct allocation for patients to receive emergency service in the best suitable time according to the severity of their condition [1, 2]. An inaccurate triage decision could prolong patients’ waiting time to use the service, which potentially leads to adverse events [2]. Various triage systems are developed to help healthcare providers make accurate triage decisions to minimize the incidence of adverse events, such as Emergency Severity Index (ESI) and Manchester Triage System (MTS) [4]. The effectiveness of triage systems were examined in various studies [5–8]. The results show the triage systems were reliable to identify patients’ severity. In addition, the application of a triage system on special situations were also reviewed, for instance, triage on pregnant women [9], telephone triage [10], and triage in low- and middle-income countries [11]. The ultimate goal of the studies is to minimize the incidence of adverse events in ED.

Triage accuracy is counted as the nurse and expert allocated the patients in the same level of triage [12]. But the concept of triage accuracy is slightly different from the current studies of reliability. The reliability of a triage system includes all kinds of inter-rater agreement among nurse-nurse and nurse-expert [5–8, 12]. And triage accuracy only counts the nurse-expert agreement. On the other hand, the validity of a triage system is determined by patient outcome that the system can ‘truly’ identify the ‘sickness’ [12]. It is different from the aim of triage accuracy to determine the time to provide interventions [1]. In brief, triage accuracy is different from reliability and validity.

With the function of triage, the incidence of adverse events in ED can be lowered with an accurate triage decision. The low incidence rate is an indicator of good quality of emergency service. In turn, a higher triage accuracy represents a better quality of emergency service. Up to current knowledge, there is no literature review examining the triage accuracy from studies. Since the triage was commonly conducted by nurse [1], the nurse-performed triage accuracy and examination method will be explored in the present review. After that, the influence of triage training is focused and followed with suggestions on practice and research to improve the triage accuracy in the future.

## Methods

**Fig. 1:**
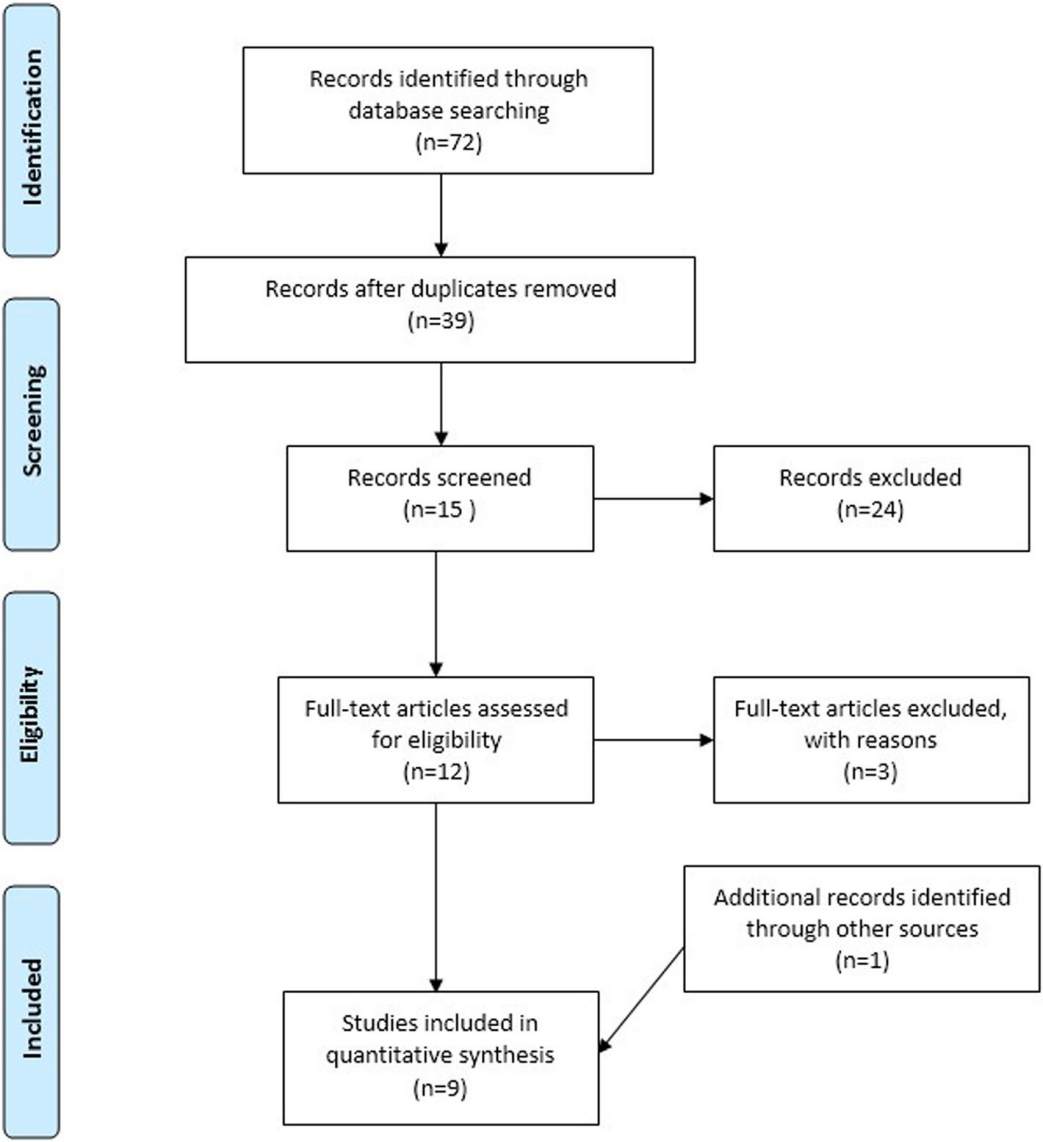
Search flow diagram

A literature search was conducted in four electronic databases, CINAHL Complete, MEDLINE with Full Text, PsycINFO and EBSCO Discovery Service, Fig. 1. The review covered a 10-year period from 2008 January to 2018 August. Keywords included ‘emergency nursing’ and ‘triage accuracy’. Thirty-nine articles were reviewed after removal of duplications. Quantitative studies written in English related to hospital triage were included. Any disaster triage, triage of special population, triage of specific condition or qualitative studies were excluded.

## Results

Eight studies written in English with full-text were identified primarily from the databases. Additional hand search of the reference of each study was conducted. A total of nine studies were included in this review as shown in Table 1. With the different triage systems used in different countries and EDs [4], the triage system used in each study is supposed to be reliable since the evaluation of triage system is out of the scope of this paper.

**Table 1:** Studies related to triage accuracy in chronological order

Study	Purpose	Design	Sample	Major findings
Olofsson et al. (2009) [13]	To examine the inter-rater reliability and triage accuracy of the triage system	Multi-center Cross-sectional study Written case scenario	79 nurses from 7 EDs Sweden	Triage accuracy: 73% The system had a good inter-rater reliability
Chen et al. (2010) [14]	To examine the triage accuracy and explore the influencing factors	Multi-center Cross-sectional study Written case scenario	279 nurses from 14 EDs Taiwan	Triage accuracy: 56.2% Influencing factors: triage training and work experience
Dalwai et al. (2014) [17]	To examine the reliability and accuracy of triage system	Single-center Cross-sectional study Written case scenario	15 nurses from one ED Pakistan	Triage accuracy: 70.1% The system cannot identify some severe cases accurately.
Martin et al. (2014) [19]	To explore the relationship between triage accuracy and work experience, triage accuracy and attitude toward patients	Multi-center Retrospective review	64 nurses from 3 EDs 1644 episodes were reviewed United States	Triage accuracy: 58.7% Neither experience or attitude was related to triage accuracy
Jordi et al. (2015) [15]	To examine the triage accuracy in German-speaking region To verify the interrater agreement across EDs To assess nurses’ confidence in using the triage system	Multi-center Cross-sectional study Written case scenario	69 nurses from 4 EDs Switzerland	Triage accuracy: 59.6% Interrater agreement: 0.78 Confidence level: 85.5%
Goldstein et al. (2017) [20]	To examine the triage accuracy and explore the reasons for inaccuracy	Single-center Retrospective review	1091 episodes were reviewed South Africa	Triage accuracy: 68.3% Discriminator errors and miscalculations were identified as major reasons of inaccuracy
Hinson et al. (2018) [22]	To examine the triage accuracy To explore the influencing factor	Single-center Retrospective review	96,071 episodes were reviewed Brazil	Triage accuracy: 82.9% Age, vital signs and chief complaints were influencing factors
Mistry et al. (2018) [16]	To examine the triage accuracy in 3 countries. To explore the relationship between accuracy and work experience	Multi-center Cross-sectional study Written case scenario	One ED in each country United Arab Emirates: 35 nurses Brazil: 30 nurses United States: 22 nurses	Overall triage accuracy: 59.2%, United Arab Emirates (58.7%), Brazil (58.3%), United States (61.3%) No relationship between triage accuracy and work experience
Rahmani et al. (2018) [21]	To examine the triage accuracy and determine the number of inaccurate cases	Single-center Retrospective review	750 episodes were reviewed Iran	Triage accuracy: 76.9% 173/577 triage cases were assigned inaccurately

Two methods were commonly used to examine the triage accuracy, written case scenario and retrospective review. The triage accuracy in each method will be shown as follows.

### Written case scenario

The method of written case scenario was used in five studies. Oloffson and colleagues examined the triage accuracy in Sweden [13]. Seventy-nine nurses from 7 EDs using MTS participated in the study. They were asked to assign the triage allocation according to the severity. There were a total of 13 examined written case scenarios. The scenarios were validated by emergency experts (two experienced physicians and three experienced nurses). The triage accuracy was 73, 13% of triage case was assigned to a less severe level than it should be. Interestingly, the overall unweighted inter-rater agreement between nurses was 0.61, which was lower than the triage accuracy [13]. This implied the individual difference on the understanding of the triage system. The difference might be related to the structure and content of triage training and it will be explored in the discussion section.

Chen and colleagues carried out a cross-sectional survey study to examine the triage accuracy in Taiwan [14]. All invited EDs used the same triage system (Taiwan triage system). 279 participants from fourteen EDs completed the survey. Ten self-developed written adult emergency case scenarios were used to examine the triage accuracy. The scenarios were piloted and demonstrated a good reliability and validity. Results showed that the overall triage accuracy was 56.2, 24.3% of inaccurate triage decision was assigned to a less severe level [14]. In the same vein, Jordi et al. used the written case scenarios to examine the triage accuracy in Switzerland [15]. They invited the triage nurses from four EDs using ESI, and 69 of them participated in their study. They extracted 30 standardized case scenarios from the official ESI implementation handbook as a tool to examine the triage accuracy. The standardized case scenarios were claimed to be reliable to examine the triage accuracy. They found the triage accuracy was 59.6, and 26.8% of the inaccurate triage decision was placed in a less severe level [15]. Their results were similar to Chen et al. findings that the severity in about half of the patients was not identified in triage, and one-fourth of the severity of triage cases was underestimated.

Mistry and colleagues conducted an international study to examine the triage accuracy [16]. They used 25 written standardized reliable and validated triage case scenarios from the ESI official implementation handbook to examine the triage accuracy in three countries, Brazil, the United Arab Emirates and the United States. They invited one hospital in each country and a total of 87 nurses from three countries participated in their study. ESI was the triage system used in these countries. Mistry et al. found the overall triage accuracy was 59.2%. No statistical difference in triage accuracy was noted between countries. In addition, the overall underestimated cases accounted for 27.6%, ranged from 24.7 to 28.9% [16]. Except for Oloffson study, the multi-center studies employing written case scenarios revealed an average triage accuracy of 58%, while the under-estimation in triage was 26% [14–16].

However, a different result was noted in a single-center study. Dalwai et al. used 42 examined written case scenarios to assess the triage accuracy in Pakistan [17]. They invited all nurses in an ED and 15 of them agreed to participate in their study. South African Triage Scale (SATS) was used in the study. The scenarios were employed from previous study, which were reaching 80% consensus in the Delphi process [18]. The triage accuracy in Dalwai et al. study was 70.1%, while 18.6% of triage cases was inaccurately put into a less severe triage level [17]. When comparing with the multi-center studies [14–16], the single-center study yielded a better triage accuracy on using written case scenarios [17]. This revealed there was some inconsistent understanding of triage system among nurses in different EDs. The issue will be explored in the discussion section. In turn, the remaining studies used the retrospective method to examine the triage accuracy.

### Retrospective review

Retrospective review was claimed to demonstrate the authentic real life triage nurse’s performance in ED. Martin and colleagues invited nurses from three EDs in Pennsylvania to participate in their study [19]. There were 64 participants and a total of 1644 episodes of triage were reviewed by six clinical expert nurses. The triage system used in their study is ESI. The triage accuracy was calculated as unweighted agreement between clinical expert nurse raters and nurse participants. The accuracy was 58.7%, but further information on inaccurate triage decision was not provided [19]. This was the only multi-center study using retrospective review to examine the triage accuracy.

Among the single-center retrospective studies, Goldstein and colleagues invited an experienced ED physician and a triage researcher to form an expert panel to review 1091 episodes of triage in an ED in South Africa [20]. The triage accuracy of SATS used in the study was 68.3 and 17.6% of episodes was underestimated. Rahmani et al. used the same method to review the triage accuracy in a teaching hospital in Iran, a single-center study [21]. They used ESI to triage the patient in ED. Three experts of emergency medicine specialists reviewed 750 episodes of triage, the accuracy was 76.9%. Only 12% of patients’ severity was under-estimated. Another single-center study was conducted in an academic hospital in Brazil. Hinson and his colleagues collected 96,071 episodes of triage to examine the accuracy [22]. ESI was used and each triage episode was reviewed by the treating emergency physician to identify whether the patient was triaged accurately. Their study yielded a high triage accuracy, 82.9%. The rate of under-estimated triage episode was only 8.8%. The triage accuracy from three single-center studies was good [20–22], which might be related to the advancement of technology to assist the decision-making in triage [23, 24]. For instance, an alert for re-consideration in triage system is shown when the input data is out of the normal range.

Either written case scenario or retrospective review has its limitation. For written case scenario, the participants cannot have further information or cue to facilitate their triage decision [14, 17]. In turn, the retrospective review is time-consuming and the raters should achieve a mutual agreement before the review. Only few studies demonstrated the characteristics of inaccurate triage decision, non-trauma cases and pediatric cases were more likely to be under-estimated [15, 16, 20]. The results are in same vein with Stanfield’s integrative review that patient characteristics could influence the decision making in triage [3].

When comparing with all multi-center studies, both methods revealed a triage accuracy of about 60% [13–16, 19] and about 23% of cases was under-estimated [13–16]. There is no standardized acceptable triage rate for all patients, but the American College of Surgeon suggested the acceptable rate of under-estimated triage episode for trauma patients was 5% and the acceptable rate of over-estimated was 25–35% [25]. If the criteria was applied on the included studies, none of the results was acceptable. Although it might not be suitable to apply the same criteria on non-trauma cases, an urge of triage improvement is needed. Training is suggested to be the most important method to improve and maintain a high level of triage accuracy [3, 14, 21].

## Discussion

All participants from the above studies had completed a triage training prior to the study, which was provided by the hospital they worked [13–17, 19–22]. Among all studies included in this review, Hinson et al. study yielded the highest level of triage accuracy [22]. The data collection period of their cross-sectional study was over two years, which might be beneficial to ED nurses to achieve a high level of triage accuracy. The reason was that the refresher triage training was provided in between, which might potentially improve the nurses’ triage performance to get a high level of triage accuracy [22]. Their results highlighted the time to provide refresher training was influential in triage accuracy.

### Structure and content of training

The inter-rater agreement among nurses and triage accuracy showed in Oloffson et al. study revealed nurses from EDs had a rather low consistent understanding of the triage system than actual triage performance [13]. In other words, nurses understood little of the triage system but coincidentally put the patients into a correct triage allocation. The coincidental allocation could place patients at risk to develop adverse events. Therefore, the triage training should be simplified to assure all ED nurses understand the use of triage system.

On the other hand, two EDs in Jordi et al. study claimed to have regular refresher triage training for ED nurses. But their triage accuracy had no statistical difference to those EDs without regular refresher training [15]. This showed that a timely manner to provide refresher training was not enough to improve the triage accuracy. The structure and content of the training should be concerned.

Brosinski and colleagues collected a 3-month triage data as baseline before training [26]. After that, they conducted four different training sessions over a month. Each session was about 20 min and consisted of 10 slides of Power Point to present different characteristics of the triage system. When all training sessions were completed, they collected another 3-month triage data and found the triage accuracy was improved significantly [26]. The divided training sessions helped ED nurses understand the triage system easily. This structured and simplified method helps deliver the content easily to make nurses to have a consistent understanding.

### Understanding of triage system

In line with the issue of inconsistency in written case scenario (Oloffson et al. study), the same issue was noted in retrospective review. Single-center study yielded a better triage accuracy than multi-center study [19–22]. A concern of the qualification of the triage instructor was raised. Two latest multi-center studies using written case scenarios showed the inter-rater agreement among nurses was higher than the triage accuracy (78%/59.6% in Jordi et al. [15] and 73%/59.2% in Mistry et al. [16]). The high inter-rater agreement revealed the understanding of triage system was similar among nurses [27]. Although their understanding about the system was rather good, the moderate level of triage accuracy showed the ED nurses did not perform well in triage. In turn, the ED nurses had common misunderstanding about the triage system. The misunderstanding might be caused by the instructors. For instance, the instructor might have misunderstanding of some aspects of the triage system and deliver them to the participants. As a result, the ED nurses demonstrated the common mistakes in some triage scenarios.

In addition, there might be some individual misunderstanding between instructors. They delivered these individual misunderstanding to the ED nurses in their workplace. Therefore, the common mistakes of triage system might not be revealed in single-center study, but the mistakes were multiplied when comparing with other EDs under the same standardized triage system. As a result, the triage accuracy from multi-center study was usually lower than that from single-center study. So, it is necessary to minimize the individual differences between instructors.

### Future direction

Refresher training is necessary for ED nurses to improve the triage accuracy. A very limited number of research studies on triage training were found to improve triage accuracy. The method used in Brosinski et al. study helps improve the accuracy [26]. Their method was easy to implement and the outcome was explicit. Although all nurses in the ED participated in the study, the total number of participants was only 15. Each session in their study was repeated 4 times. As a result, each instructor spent 5 h 30 min to complete all four sessions for 15 nurses [26]. Their method might be time-consuming if the number of ED nurses increases [28]. Further study is suggested to examine whether this method can be applied effectively in ED with more nurses. Various methods such as online training [29, 30] and human patient simulation [31, 32] were developed to improve triage accuracy. But the effectiveness of these methods needs to be evaluate with more participants in future study.

On the other hand, the ED with regular refresher triage training and high triage accuracy (e.g. the ED in Hinson et al. study [22]) can develop a structured training program for other EDs. This can minimize the inconsistent triage level between EDs in the same country. In other words, this can help improve the quality of triage performance within the country, and potentially lower the incidence of adverse events in ED.

In addition, a network collaboration between EDs can be established to discuss and provide suggestions on the use of triage system within the country. The network helps clarify any misunderstanding of the triage system, and a structured triage training with consistent content can be developed for all EDs within the country. The qualification of triage training instructor can be formulated through the discussion in the network. Furthermore, the network can provide suggestions for the research team of the triage system to improve the development of the system in future. And the network can set an acceptable triage rate for the country to improve the overall triage performance.

Lastly, a continuous monitoring is important. Every ED can set their own standard of acceptable triage rate if a mutual agreement within the country is not achieved. A regular monthly surveillance might help recognize a deterioration of triage rate in an early stage [21, 26]. The triage accuracy can be formulated by comparing the physician-assigned triage decision with nurse-assigned triage decision as used in Hinson et al. study [22]. This method is fast and convenient to get the result within a short time, but it is difficult to achieve a mutual agreement if the number of physicians is increased. A surveillance team as in Martin et al. study is suggested [19]. A small number of members can achieve the mutual agreement easily. Since the triage accuracy showed a significant difference in various work shifts [21], equal proportion of cases should be extracted from each shift if triage cases are randomly selected for surveillance. In addition, the authentic inaccurate triage cases can be collected and integrated into the triage training. But the patient confidentiality should also be treated carefully. Further studies are suggested to explore other appropriate methods to review the real case triage accuracy, while maintaining the patient confidentiality.

## Conclusion

An accurate triage decision is essential for patients to receive the emergency service in the most appropriate time. Triage accuracy is different from reliability and validity, that only the nurse-expert agreement is counted. This review identified two common methods to examine the triage accuracy, and found the accuracy was in a moderate level in general. However, a study conducted in one hospital showed a rather good result. This might be related to the regular refresher triage training.

The triage training should be structured and provided by an instructor, who is very familiar with the triage system. A collaboration between EDs is suggested to affirm the same patient is allocated in the same triage level in any ED of the country. A monthly surveillance is suggested and the acceptable triage accuracy should be formulated to identify the needs of triage training at the earliest time. An early recognition of triage inaccuracy facilitates an early intervention to minimize the incidence of adverse events in ED. As a result, the overall triage performance can be improved.

Since there are limited studies focusing on triage training, further studies are suggested to explore and examine the effectiveness of triage training to improve triage accuracy.

## Abbreviations

e.g.: for example
ED: emergency department
ESI: Emergency Severity Index
MTS: Manchester Triage System
SATS: South African Triage Scale
